# Efficient Exfoliation of Layered Double Hydroxides; Effect of Cationic Ratio, Hydration State, Anions and Their Orientations

**DOI:** 10.3390/ma14020346

**Published:** 2021-01-12

**Authors:** Jayakumar Karthikeyan, Helmer Fjellvåg, Silje Bundli, Anja Olafsen Sjåstad

**Affiliations:** Department of Chemistry, Centre for Materials Science and Nanotechnology, University of Oslo, Post Office Box 1033, 0315 Oslo, Norway; jayakumar.karthikeyan@smn.uio.no (J.K.); helmer.fjellvag@kjemi.uio.no (H.F.); siljeanbu@gmail.com (S.B.)

**Keywords:** layered double hydroxides, exfoliation, morphology, nanosheets

## Abstract

The exfoliation ability of nitrate based Mg_1−*x*_Al*_x_*(OH)_2_(NO_3_)*_x_*·*m*H_2_O layered double hydroxides (Mg-Al LDH) in formamide into single or multilayer nanosheets depends strongly on nitrate anion orientation and layer charge. Our systematic studies used materials that were likely to disclose differences with respect to anion type and their concentrations in the interlayer gallery. We assured to avoid any carbonate incorporation into the galleries for nitrate, chloride, iodide, and sulfate based Mg-Al LDHs. Furthermore, the comparative exfoliation experiments were conducted for fully hydrated samples with as similar particle morphology as possible. The exfoliation of nitrate Mg-Al LDH is far superior to similar clays with carbonate, sulfate, chloride, or iodide as charge balancing anions. Quantitative analysis of exfoliation yield for pre-treated, fully hydrated samples, shows an optimum composition for exfoliation into single nanosheets of around *x* ≈ 0.25, while double or triple layered sheets are encountered for other *x*-values. We observe a clear correlation between the expansion of the interlayer gallery due to progressing tilts of nitrate anions and water molecules out of the horizontal interlayer plane, suspension turbidity, and degree of exfoliation. The established correlations extends to nitrate Ni-Al LDH materials. We finally claim that morphology is a dominating parameter, with house-of-card morphology particles exfoliation far less than platelet-like particles. Hence, hydrothermal treatment may be favorable to enhance exfoliation yields.

## 1. Introduction

Low dimensional solids find wide applications based on their chemical, electronic [1] photonic [2], magnetic [3], and mechanical properties [4]. Among these, the layered double hydroxides (LDH), [*M*^II^_1−*x*_*M*^III^*_x_*(OH)_2_][*A^n^*^−^]*_x/n_*·*m*H_2_O, are intensively studied as minerals and as synthesized compounds with highly tunable compositions. Their atomic arrangement is related to that of brucite, and the incorporation of higher valency cations. *M*^III^ is charge compensated by anions (*A^n^*^−^) in the interlayer gallery along with water molecules. Their stability covers at least the range 0.20 ≤ *x* ≤ 0.33 [5]. The compensating anion (*A^n^*^−^) spans from small inorganic to complex organic species [5]. The thermodynamic stability and solubility of LDHs based on different anions vary as NO_3_^−^ < SO_4_^2^^−^ < CO_3_^2^^−^; likewise the anion exchange ability NO_3_^−^ < Cl^−^ < SO_4_^2^^−^ < CO_3_^2^^−^ [6]. A high surface area for the precipitated LDHs reflects a small particle size of platelet or house-of-card morphology. Their good ion exchange capacity makes LDHs applicable as, e.g., absorbents and additives. A fascinating aspect with certain two-dimensional (2D) materials is their ability to exfoliate into single (or multiple) layered nanosheets, and/or crystallize as tubes or scrolls. In this respect, LDHs can be exfoliated, providing single 2D nanosheets of < 1 nm thickness that may serve as building bricks for functional nanocomposites with positively charged backbones, compared to silicates with negatively charged backbones [7,8,9,10].

In a strive to optimize exfoliation, preferably in formamide, various factors have been addressed in the past; e.g., chemical nature and composition of the 2D-brucite like layers [11,12], anion/solvent pair [13,14], drying procedure [11,15], water content [16], charge of anions [16], hydrothermal treatment, particle morphology, and mode of drying [12]. A challenge for all systematic studies is sufficient control on the interlayer chemistry, stacking faults, and polytype variants inclusive. The layer charge correlates directly with the *M*^III^ content and affects the interaction strength with the polar formamide solvent molecules. For instance, Hibino and Jones [11] found that Mg_1−*x*_Al*_x_*(OH)_2_(glycinate)*_x_*·*m*H_2_O *x* = 0.25 exfoliates better than *x* = 0.33 and *x* = 0.20 in formamide.

Molecular dynamics (MD) simulations are highly interesting, but may have limitations since certain material properties related to the atomic arrangement are defined at the nucleation/growth stage of the LDHs, and subsequent transformations may not occur in reality—although predicted by energetics, yet hindered by kinetics [17]. Similar obstacles caused by slow kinetics may apply to interlayer exchange reactions. It remains open whether MD modulations can fully predict properties of house-of-card interconnected particles, or whether they rather best apply to samples with platelet morphology. Nevertheless, MD simulations and experiments [18,19] agree that concentration and orientation of interlayer nitrate anions have major effects on the gallery space. During the exfoliation process, the intercalated solvent/surfactant molecules connect to interlayer anions and hydroxyls of the brucite-like layers by hydrogen bonding, as well interact with the positively charged 2D LDH sheets through electrostatic interactions [13]. At a low layer charge, the gallery space is tight with flat lying nitrate anions (D_3h_ symmetry; threefold axes perpendicular to LDH-layers). At high(er) layer charges, the nitrate anions and water molecules are tilted out of the horizontal plane, causing expansion of the *c*-axis [19]. Hence, the *c*-axis is a measure of the nitrate orientation. However, hydration also triggers expansion, yet much less, and can be tuned by temperature, relative humidity, and holding time. The latter effect is strongest at intermediate *x* compositions; e.g., *x* = 0.25. Simulations indicate that nitrate anion reorientation may be triggered by very high water contents, however, such contents are, to our knowledge, not achieved experimentally [20].

The current work builds on ref. [12] and our insight into LDH materials [8,9,12,19,21], which allow us now to address questions where high control of chemistry and morphology is needed to disclose correlations with ability of exfoliation. We focus on how the cationic ratio (layer charge) influences nitrate anion orientation, and in turn also the exfoliation properties of Mg_1−*x*_Al*_x_*(OH)_2_(NO_3_)*_x_*·*m*H_2_O, *x* = 0.12–0.42. Since morphology has a major effect on exfoliation, we maintain a house-of-cards like morphology to allow systematic comparisons. Likewise, we restrict ourselves to fully hydrated LDHs. We use the nitrate–formamide pair as the best choice for efficient exfoliation according to Sasaki et al. [13] and Wu et al. [14]. A robust procedure for quantifying the degree of exfoliation [12] is adopted. The current experimental study benefits from recent simulation works, where molecular dynamics have been used to analyze the effect of cation ratio (layer charge), type of anion, and water content on polytypism, interlayer structure, and swelling behavior. Furthermore, very useful structure and stacking fault simulations provide means to facile interpretation of powder X-ray diffraction data for different situations with respect to stacking faults, nanosheet thickness, water content, etc. 

We argue that the nitrate based *x* ≈ 0.25 composition is optimal for successful exfoliation and that this composition (and corresponding layer charge) will enable benchmarking of the exfoliation behavior of nitrate based samples in comparison with less performing Cl^−^, I^−^, CO_3_^2^^−^, and SO_4_^2^^−^ based Mg-Al LDH systems. We show that the chosen synthesis condition is an important parameter owing to incorporation of OH^−^ at higher pH. In particular, we address how the orientation of the nitrate ion and thereby the gallery space correlates with exfoliation ability, and discuss this behavior in light of corresponding data for the Ni_1−*x*_Al*_x_*(OH)_2_(NO_3_)*_x_*·*m*H_2_O system.

## 2. Materials and Methods 

### 2.1. Synthesis and Characterization

Samples of Mg_1−*x*_Al*_x_*(OH)_2_(NO_3_)*_x_*·*m*H_2_O, *x* = 0.12–0.42 were synthesized by co-precipitation at 60 °C and pH = 10.0 under strict atmospheric control [12,14,21]. Correspondingly, *x* = 0.20, 0.25, and 0.33 were synthesized for *A^n^*^−^ = Cl^−^, I^−^, CO_3_^2^^−^ and SO_4_^2^^−^. Additional syntheses at pH = 11.0 were done for nitrate samples. Chemicals from Sigma-Aldrich were used; Mg(NO_3_)_2_·6H_2_O (99%), Al(NO_3_)_3_·9H_2_O (98%), MgSO_4_ (99.5%), Al_2_(SO_4_)_3_·H_2_O (98%), MgCl_2_ (98%), AlCl_3_·6H_2_O (99%), MgI_2_ (98%), and AlI_3_ (95%). Efforts were taken to minimize carbonate contaminations; deionized water was boiled during He-bubbling; KOH pellets were stored inertly; KOH solutions were prepared in argon tent; all vessels were purged with He gas during reactions. Typically, the reaction vessel was filled with 100 mL degassed water and a mixture of a few mL of ≈0.85 M KOH, and 0.15 M KNO_3_ solution was added with a peristaltic pump until target pH was reached. 100 mL of the cation solution was added at a constant rate (1.25 mL/min) via a peristaltic pump. pH was maintained constant by feedback to a pH titrator. The suspension was aged for 90 min, washed four times in a closed container, and freeze dried. Ni_1−*x*_Al*_x_*(OH)_2_(NO_3_)*_x_*·*m*H_2_O samples were synthesized similarly, at pH = 9.0 and 10.0 using Ni(NO_3_)_2_·6H_2_O (99%) as an additional precursor. Mg_1−*x*_Al*_x_*(OH)_2_(NO_3_)*_x_*·*m*H_2_O and Ni_1−*x*_Al*_x_*(OH)_2_(NO_3_)*_x_*·*m*H_2_O samples are throughout the text denoted Mg-Al LDH and Ni-Al LDH, respectively.

All products were characterized by powder X-ray diffraction (XRD; G670 Huber Transmission diffractometer; CuKα_1_ radiation, λ = 1.54056 Å; 0.7 mm glass capillaries) using silicon (*a* = 5.4309 Å; NIST) as the internal standard. Unit cell dimensions (*R*$\bar{3}$*m*, hexagonal setting) were calculated from (110) and (00*l*) according to *a* = 2 *d*_110_ and *c* = [3 × *d*_003_ + 6 × *d*_006_]/2. Cationic compositions were determined by Microwave Plasma Atomic Emission Spectroscopy (Agilent 4100 MP-AES; estimated uncertainty in *x* composition ± 0.01 units). CN analysis was performed at Ilse Beetz Microanalytisches Laboratorium, Germany. Transmission electron microscopy (TEM) images were obtained using a JEOL 2010F operating at 200 kV. 

### 2.2. Pre-Treatment, Exfoliation, Quantification and Characterization

Standardized pre-treatments were done to achieve a saturated hydration level of the as-synthesized Mg_1−*x*_Al*_x_*(OH)_2_(NO_3_)*_x_*·*m*H_2_O, Ni_1−*x*_Al*_x_*(OH)_2_(NO_3_)*_x_*·*m*H_2_O and Mg_1−*x*_Al*_x_*(OH)_2_(*A^n^*^−^)*_x_*_/*n*_·*m*H_2_O (*A^n^*^−^ = Cl^−^, I^−^, CO_3_^2^^−^ and SO_4_^2^^−^) powders prior to exfoliation studies. Samples were exposed to humidity in closed containers filled with a few mL of degassed water and argon. Exfoliation in formamide was thereafter performed immediately under ultrasonic treatment for 4 h [12]. Two hundred mg LDH was dispersed in 20 mL formamide in glass cuvettes that were rotated (1 rpm) in the sonication bath (VWR, USC600D) at 20–25 °C to ensure identical conditions. The turbidity was measured in nephelometric turbidity units (NTU) using a Merck Turbiquant 3000 IR. After sonication, the suspensions were centrifuged at 19,600× *g* (rcf) for 10 min. Owing to full correspondence between small angle X-ray scattering (SAXS) and atomic force microscopy (AFM) data for exfoliated nanosheets in suspension [12,14,21] just AFM was currently applied (at atmospheric conditions; Nanoscope E multimode AFM in tapping mode; silicon etched probe with three rectangular cantilevers). The degree of exfoliation was quantified following our already reported four step gravimetric method [12]. The analysis is based analysis of the calcined and dry sediments obtained after centrifugation of the exfoliation suspensions (as described above); i.e., the large particles classified as not exfoliated and were obtained as sediment by applying rcf = 19,600 g for 10 min. In the procedure, it is essential to ensure complete removal of formamide and to preheat the porcelain crucibles at 900 °C to constant mass prior to use. In addition, empty and loaded crucibles were transferred at 900 °C to a desiccator for cooling and storage. For details regarding calculations and formulas, consult [12]. Sample preparations were done in a class 100,000 clean room.

## 3. Results

### 3.1. Materials; Hydration, Morphology and Effect of Anions

The Mg-Al LDH-nitrate samples, 0.12 ≤ *x* ≤ 0.42, synthesized at CO_2_ free conditions, enabled us to study correlations between cation/anion concentration (layer charge), anion type, and the ability of exfoliation. For all Mg-Al LDH samples, (*A^n^*^−^ = NO_3_^−^, Cl^−^, I^−^, CO_3_^2^^−^, SO_4_^2^^−^), we note a good correspondence between nominal and average product composition as measured by MP-AES, Table 1 and Table 2. With focus on the Mg-Al nitrate samples; XRD shows the compounds to be phase pure, except *x* = 0.12 with trace amounts of Mg(OH)_2_, and *x* = 0.17 with MgO. Minor amorphous fractions cannot be ruled out. CN elemental analysis reveals products with a very high nitrate to carbonate ratio of N:C ≈ 14:1 [*x* = 0.20, N content 2.9 wt.% (theoretical: 2.8 wt.%); *x* = 0.25, N = 3.5 wt.% (theoretical: 3.5 wt.%); *x* = 0.33, N = 4.3 wt.% (theoretical: 4.6 wt.%)]. The low carbon level for the as-synthesized LDH is quite equal to that of the Mg-Al oxide calcined at 480 °C for 24 h. Probably, very minor amounts of carbonate is absorbed at surfaces during unavoidable exposure to low, yet non-zero, levels of air/moisture [19].

The XRD data provide important insight to the layered compounds, see some representative compositions in Figure S1. The broad (003) and sharp (110) peaks are consistent with interlayer height variations [19] and stacking disorder of otherwise well-defined sheets. The FWHM of (003) is largest for *x* = 0.25, which is probably a signature of a broader distribution of water and nitrate tilt angles out of the interlayer plane [19], but possibly also with respect to spatial variations in the water content.

**Figure 1:**
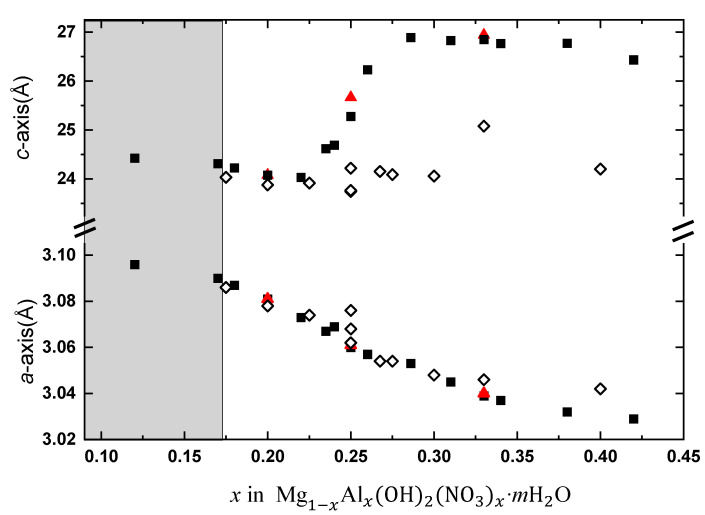
Variation of the *a*- and *c*-axis with *x*, Al(III) content, for as-synthesized (■) and post hydrated (▲) Mg_1−*x*_Al*_x_*(OH)_2_(NO_3_)*_x_*·*m*H_2_O layered double hydroxides (LDHs) synthesized at pH 10.0. (◇) represent synthesis at pH 11.0. Gray shading indicate range where additional diffraction peaks prove lack of phase purity, see text.

The variation in unit cell dimensions (*a*, *c*) is shown in Figure 1; for details see Table 1. At low Al^III^ and nitrate contents, a short *c*-axis that is slightly contracting with increasing layer charge is consistent with flat lying anions (D_3h_ symmetry). The onset of a major *c*-axis expansion at around *x* = 0.23 (pH = 10.0) correlates with a progressing reorientation of the nitrate anions and water molecules as shown by modeling [19]. Correspondingly, the contraction for *x* > 0.28 is due to electrostatic interactions between the positive brucite-like layers and negatively charged species in the gallery. The *a*-axis decreases quite linearly when smaller Al^III^ atoms substitute for Mg^II^ in the edge shared octahedra of the solid solution. The *a*-axis is similarly contracting for Cl^−^, I^−^, CO_3_^2^^−^, and SO_4_^2^^−^ LDHs, Table 2. Data for the analogous Ni-Al nitrate LDH solid solution is shown in Figure S2. 

We observe that for synthesis at high pH, the nitrate anions are (partly) replaced by hydroxide anions with Mg_0.75_Al_0.25_(OH)_2_(OH)_0.25_·0.5H_2_O [Mg_6_Al*_2_*(OH)_18_·4H_2_O; Meixnerite]; *a* = 3.0463 Å, *c* = 22.93 Å, representing the end point of the likely NO_3_^–^-OH^−^ solid solution. The small OH^−^ anions do not cause a molecular reorientation like the nitrate anions, and hence there is no major *c*-axis expansion, see Figure 1 (pH = 11.0), and they behave in this respect similarly to chloride ions. There are hence delicate balances to be respected during synthesis; lack of inert conditions gives carbonate impurities, whereas too high pH leads to hydroxyl incorporation. For Ni-Al nitrate LDHs synthesized at pH = 9.0, we note a similar expansion of the *c*-axis in exactly the same *x*-composition interval as for the Mg-Al nitrate samples, also due to nitrate and water out-of-plane tilting. At pH = 10.0, the expansion is less, which again reflects incorporation of hydroxide anions to the interlayer gallery, Figure S2.

**Figure 2:**
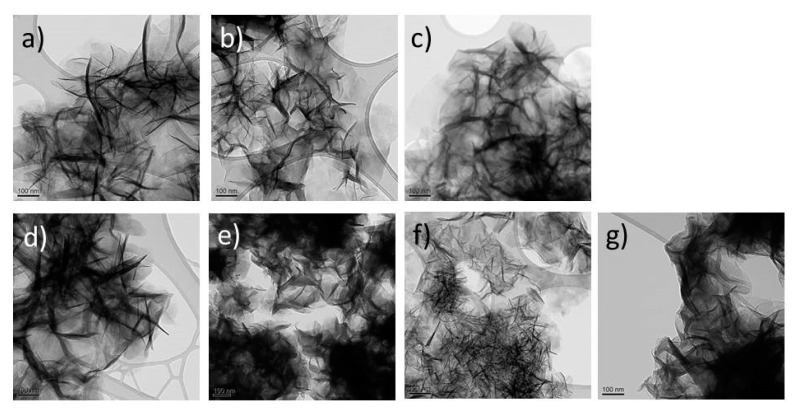
Representative TEM images of selected Mg_1−*x*_Al_x_(OH)_2_(*A^n^*^−^)*_x_*_/*n*_·*m*H_2_O LDHs. *A^n^*^−^ = NO_3_^−^, *x* = (**a**) 0.20, (**b**) 0.25, (**c**) 0.33. *A^n^*^−^ (*x* = 0.25), (**d**) Cl^−^, (**e**) I^−^, (**f**) CO_3_^2^^−^, (**g**) SO_4_^2^^−^. All samples shows the typical house-of-card morphology, and the particles have similar platelet thickness and size.

Although MD modeling has shown that very high water contents lead to substantial expansion and even polytype transformations [22], we do not observe any such effects experimentally. The as-received freeze dried powders (Table 1 and Table 2) have low water contents as a result of the applied dynamic vacuum, and have hence short *c*-axes, Table 1. The representative TEM images in Figure 2a–c show platelet like Mg-Al LDH crystallites oriented at random with a house-of-card morphology. The particle diameter is approximately 100 nm for the analyzed Cl^−^, I^−^, CO_3_^2^^−^, and SO_4_^2^^−^ LDHs (Figure 2d–g). A similar morphology is observed for the current nitrate based Ni-Al LDHs, however, with a smaller particle size (platelet diameter/thickness: Mg-Al LDH 100–250 nm/6–10 nm; Ni-Al LDH 25–40 nm/< 5 nm) [12].

Data on chemical composition, layer charge, unit cell dimensions, and exfoliation properties of Mg_1−*x*_Al*_x_*(OH)_2_(*A^n^*^−^)*_x_*_/*n*_·*m*H_2_O with *A^n^*^−^ = Cl^−^, I^−^, CO_3_^2^^−^, SO_4_^2^^−^ are listed in Table 2. The height between layers in terms of the *d*-value for (003) of 7.8 Å is characteristic for the carbonate-bearing hydrotalcite (*x* = 0.25) with flat-lying anions in the interlayer [23]. On incorporation of sulfate anions (*x* = 0.26), *d*_003_ expands to 8.7 Å, in line with findings by Miyata [24]. The spherical chloride and iodide anions (*x* = 0.25) give rise to *d*-spacings of 7.8 and 8.2 Å, respectively, consistent with Iyi et al. [23]. For the sulfate series, an initial poor packing of the tetrahedral anions with different orientations with respect to the hydrogen bonding OH-layers is likely to become more organized when the interlayer gallery becomes crowded, which results in elongation of the *c*-axis on increasing *x.* On the other hand, a contraction is observed for carbonate, chloride, and iodide. For all these Mg-Al LDH solid solutions, the *a*-axis contracts smoothly with increasing Al^III^ content.

### 3.2. Exfoliation, Quantification, and Characterization of Suspensions

**Figure 3:**
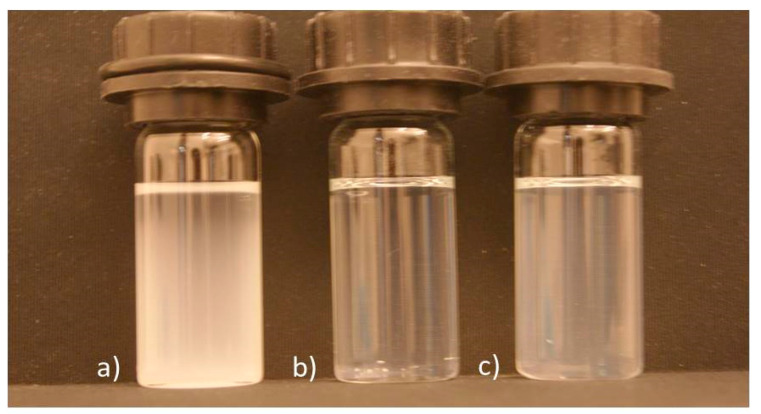
Cuvettes with exfoliated suspensions (after 4 h) of Mg_1−*x*_Al*_x_*(OH)_2_(NO_3_)*_x_*·*m*H_2_O with *x* = (**a**) 0.20, (**b**) 0.25, (**c**) 0.33. A complete comparison of difference in end turbidity between suspensions of Mg-Al nitrate LDHs versus *x* is shown in Figure S3.

For LDH particles with similar morphologies and chemical compositions, turbidity measurements can conveniently be used for monitoring the progress of exfoliation [12]. For such simplifying conditions, measured end turbidities for delamination in formamide are presented in Table 1 and Table 2. Figure 3 clearly visualizes differences in turbidity between suspensions of Mg_1−*x*_Al*_x_*(OH)_2_(NO_3_)*_x_*·*m*H_2_O with *x* = 0.20, 0.25, and 0.33.

**Figure 4:**
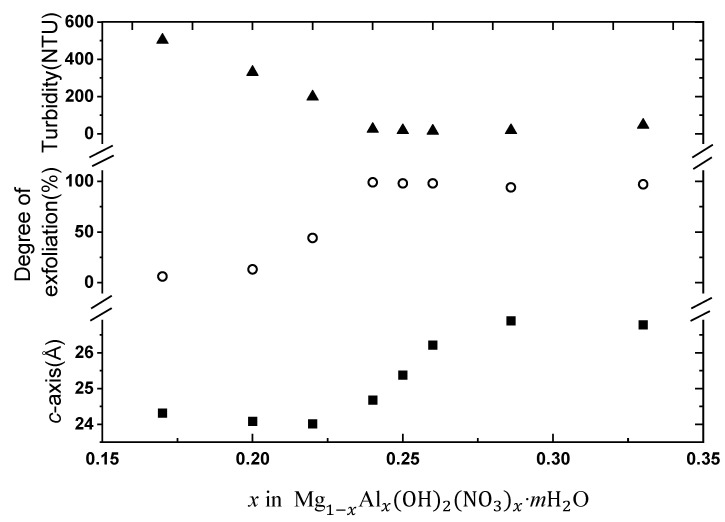
Turbidity (▲), degree of exfoliation (○), and c-axis (■) versus Al(III) content *x* in Mg_1−*x*_Al*_x_*(OH)_2_(NO_3_)*_x_*·*m*H_2_O.

The end turbidity varies from 500 NTU for Mg-rich nitrate samples to a minimum of <20 NTU for *x* ≈ 0.25, and increases again slightly (50 NTU) for higher *x*, see Table 1 and Figure 3. Mg-Al LDHs with other anions (Cl^−^, I^−^, CO_3_^2−^ or SO_4_^2−^) show high end turbidities (200–1100 NTU). The degree of exfoliation of Mg_1−*x*_Al*_x_*(OH)_2_(NO_3_)*_x_*·*m*H_2_O was quantified gravimetrically, see Table 1 and Table 2, and depends strongly on *x* [Al^III^; nitrate content] with a maximum at *x* ≈ 0.25, Figure 4. 

There is a close correspondence between the degree of exfoliation and the length of the *c*-axis (i.e., interlayer gallery height), Figure 4. However, a closer look shows that there is not a fully 100% matching between change in turbidity, *c*-axis expansion, and degree of exfoliation. For LDHs with Cl^−^, I^−^, CO_3_^2−^, or SO_4_^2−^ as interlayer anion, the degree of exfoliation was in all cases low, 15–32%, Table 2. 

Based on the expansion of the gallery on increasing nitrate content, one could by analogy to Mg-Al nitrate LDHs also expect good exfoliation properties for Ni-Al nitrate LDHs. However, we find the exfoliation properties to be inferior for Ni-Al LDHs [12], with the same house of cards morphology, yet smaller particle size.

Tapping mode AFM data for exfoliated *x* = 0.25 nitrate Mg-Al LDHs show well dispersed nanosheets with disc like morphology, 30–40 nm in lateral dimensions, and 0.70 ± 0.07 nm heights (average 10 particles). Typical tapping mode AFM images are shown in Figure S4. The height (and standard deviation) indicates a single disc thickness that corresponds to an individual nanosheet. From crystallographic data the thickness of just the brucite nanosheet is around 0.48 nm [13,16]; in addition comes the adsorption of formamide and counter anions that defines the effective thickness. For *x* = 0.20 and 0.33, the observed disc thickness is higher; 2.4 ± 0.7 nm and 2.1 ± 0.7 nm, respectively (standard deviation based on ten particle heights). The observed minor discrepancies in the compositional variation of turbidity and degree of exfoliation is possibly caused by different thicknesses of the obtained nanosheets. When considering the AFM data, the degree of exfoliation must be kept in mind. The concentration of nanosheets on the mica surface is larger for *x* = 0.25 and 0.33 than for the poorly exfoliated *x* = 0.20 sample. For *x* = 0.20, major amounts of large LDH particles settle during the high-speed centrifugation. Note that the AFM images of the nanosheets represent just a small percentage of the material that underwent the exfoliation protocol.

## 4. Discussion

Our earlier studies [12] showed that samples with severe cross-linking of the layer-like LDH particles, taking so-called house-of-cards morphology, exfoliate just to a limited extent. Smaller particle size and enhanced crosslinking may blur exfoliation features being intrinsic to the ideal LDH material free of stacking faults. 

We observe clear correlations between crystal structure and interlayer chemistry of Mg_1−*x*_Al*_x_*(OH)_2_(*A^n^*^−^)*_x/n_*·*m*H_2_O clay materials and their ability to undergo exfoliation. We identify these once other parameters are sufficiently constrained, approaching one-parameter like investigations. In this respect, control of anion purity, water saturation of interlayer gallery, as well as maintaining close similarity in morphology is essential. 

When turning to details, the average 3R_1_ polytype stacking remains to the best of our knowledge unchanged for different *M*(III) contents in the Mg-Al and Ni-Al LDH systems. However, we are aware from (unpublished) total scattering and PDF (pair distribution function) data that certain smaller, yet significant, variations remain unexplained. Hence, certain secrets are still undisclosed.

The layer charge and the *a*-axis vary more or less identically with *x* for the various anion systems. Yet, these systems differ significantly; (i) first by the anion charge; monovalent versus divalent (carbonate, sulfate) with implications on anion concentration and on electrostatic interactions towards the brucite layered backbone; (ii) second, by the anion size and geometry which influence anion orientation and thereby length of the *c*-axis, especially at high anion concentrations; and (iii) to a lesser extent, but nevertheless, the hydration level [25]. Issue (ii) is particularly important for monovalent multi-atom anions like the triangularly shaped NO_3_^−^ and ClO_3_^−^. These can be forced out of their horizontal orientation when their concentration becomes too large. Figure 1 indeed shows that the interlayer gallery widens up for *x* > 0.23 for nitrate LDHs. Such nitrate LDHs are well known to promote ion exchange, e.g., intercalation of long chained alkyl carboxylic acids into Zn-Al nitrate LDHs [26]. 

Formamide is highly polar with donor and acceptor groups. Its carbonyl function interacts strongly with the OH-groups of the LDH host. On the other hand, its amino functions are barely interacting with anions and water, unable to form a strong network of hydrogen bonds in the interlayer. The strong polar interactions between the positive backbone and formamide have been utilized for bottom-up synthesis of single LDH nanosheets [27], favored by enhanced layer charge. On increased concentration of formamide, a higher yield of nanosheets was observed. In our current comparative studies, we use, on the other hand, a standardized amount of 20 mL formamide for a 200 mg LDH sample. Any increase in the relative amount of formamide in our studies will enhance the level of exfoliation. 

Intercalation of formamide is the first stage of the exfoliation process, partly replacing water molecules and promoting swelling owing of a weakening of the hydrogen-bonding network [13]. Hence it is reasonable to postulate that variations in the degree of exfoliation is related to formamide intercalation. In the current comparative study, the concentration of water molecules filling the interlayer can be regarded as constant; furthermore, the strength of the Coulombic interaction between the positive charge of the brucite layer and formamide varies in the same way for all anion systems. Hence, major differences between the explored systems refer to (i) space for insertion as measured by the interlayer height; *c*-axis, (ii) concentration of interlayer anion and its ability to involve in hydrogen bonding network; and (iii) strength of Coulombic interaction between the brucite layer and the interlayer anions. 

Even for the well exfoliating Mg-Al nitrates (*x* = 0.25), we note that the exfoliation ability deteriorates when the interlayer gallery is tight (*x* = 0.20) and the polar interactions towards formamide are moderate owing to low layer charge. The tight gallery is maintained for chloride, iodide, and carbonate LDHs. Whereas Cl^−^ and I^−^ do not form hydrogen bonds, the situation is different for carbonate. These observations suggest that space for formamide intercalation actually is a key parameter. This is supported by the observed good exfoliation ability for amine functionalized LDHs [16]. For divalent anion LDHs, enhanced Coloumbic interactions are a likely additional parameter for poor intercalation and delamination ability. We believe that this is also the reason for lower delamination ability for high *x* nitrate samples. A higher layer charge will result in stronger interactions with the formamide agent, however, the energy balance may still stabilize the LDH compound via Coulombic interactions as indicated by thermal stability studies by TGA [19]. This is consistent with AFM images showing that *x* = 0.25 exfoliates into single nanosheets, whereas *x* = 0.33 typically gives nanosheets of 2–3 layer thickness (1.4 ± 0.7 nm).

It is interesting to note that the degree of exfoliation of Ni_0.75_Al_0.25_(OH)_2_(NO_3_)_0.25_·*m*H_2_O improves from 10% to almost fully exfoliated by means of a hydrothermal post treatment [12]. Obviously, the basic chemistry is identical between the as synthesized house-of-cards LDH and the hydrothermal platelet-like LDH. From XRD, we note that (003) sharpens considerably on hydrothermal post treatment, with just a small shift in *d*-spacing [interlayer height and *a*-axis; reflecting a tiny change in Al(III)-content]. This probably proves a more homogeneous distribution of water molecules and nitrate anions, with respect to concentration and to tilt angles. This may be caused by the larger platelet size, and the lack of interfaces towards interwoven crystallites. We suggest that the hydrothermal treatment initiates a recrystallization process towards the more well-defined LDH materials. Possibly, the genuine 3R_1_ polytype material properties are only shown for platelet like morphology, and remains hidden for house-of-card structures. We speculate whether even poorly exfoliating Mg_0.75_Al_0.25_(OH)_2_(*A^n^*^−^)_0.25*/n*_·*m*H_2_O (*A^n^*^−^ = Cl^−^, I^−^, CO_3_^2^^−^ and SO_4_^2^^−^) can possess improved exfoliation properties if optimized (and recrystallized) during hydrothermal post treatment. 

## 5. Conclusions

In summary: (i) For nitrate based Mg-Al LDHs, humidity pre-treatment has a positive effect on exfoliation, however, the improvement is small and probably not required for still achieving facile exfoliation; (ii) nitrate bearing LDHs exhibit a sufficiently high degree of exfoliation in formamide being useful for practical purposes; maximum amounts of single nanosheets are obtained for *x* ≈ 0.25; exfoliation into double or triple sheets is typically encountered for other *x*-values; (iii) the end turbidity for *x* = 0.25 samples with *A^n^*^−^ = Cl^−^, I^−^, CO_3_^2^^−^, and SO_4_^2^^−^ is consistent with poor capability towards exfoliation; (iv) modifying the morphology by hydrothermal treatment into more platelet like crystallites have a positive effect on exfoliation ability.

## Figures and Tables

**Table 1:** Composition, layer charge, unit cell dimensions, and exfoliation properties of Mg_1−*x*_Al*_x_*(OH)_2_(NO_3_)*_x_*·*m*H_2_O; *x* = 0.12–0.42.

*x* Nom.	*x* Exp.	Charge Density 10^2^ (q_e_/Å^2^)	*x*-Axis ^1^ (Å)	*d*-Spacing		*c*-Axis ^2^ Freeze Dried (Å)	*c*-Axis ^2^ Pretreat (72 h) (Å)	End Turbidity (NTU)	Degree of Exfoliation (%)	Nanosheet Diameter (AFM) (nm)	Nanosheet Thickness (AFM) (nm)
				*d*_003_ (Å)	*d*_006_ (Å)						
0.12	0.13	1.45	3.096	8.083	4.101	24.428					
0.17	0.18	2.23	3.090	8.125	4.042	24.314		504	6		
0.18	0.19	2.36	3.087	8.095	4.028	24.227					
0.20	0.21	2.55	3.081	8.054	4.000	24.081	24.113	331	13/9^4^	37	4.2 ± 0.7
0.22	0.22	2.78	3.073	8.056	3.984	24.036		199	44		
0.23	0.24	2.99	3.067	8.248	4.082	24.618		26	99		
0.24	0.24	2.99	3.069	8.276	4.093	24.693					
0.25	0.25	3.16	3.060	8.452	4.220	25.278	25.666	19	98/98^4^	32	0.75 ± 0.0
0.26	0.25	3.16	3.057	8.744	4.372	26.232		16	98		
0.28	0.27	3.46	3.053	8.962	4.483	26.892		19	82		
0.31	0.31	3.90	3.045	8.935	4.476	26.831					
0.33	0.33	4.13	3.039	8.964	4.469	26.853	26.985	48	97/97^4^	39	2.1 ± 0.7
0.34	0.33	4.23	3.037	8.940	4.452	26.770					
0.38	0.36	4.77	3.032	8.947	4.450	26.770					
0.42	0.39	5.29	3.029	8.831	4.398	26.467					

^1^*a* = 2*d*_110_; ^2^ *c* = (3*d*_003_ + 6*d*_006_)/2.

**Table 2:** Chemical composition, layer charge, unit cell dimensions, and exfoliation properties of Mg_1−*x*_Al*_x_*(OH)_2_(*A^n^*^−^)*_x/n_*·*m*H_2_O with *A^n^*^−^ anions = Cl^−^, I^−^, CO_3_^2^^−^, SO_4_^2^^−^.

Anion	Nom. Comp. *x*	Exp. Comp. *x*	Charge Density (10^2^q_e_/Å^2^)	*a*-Axis ^1^ (Å)	*d*-Spacing (Å)		*c*-Axis ^2^ (Å)		End Turbidity (NTU)	Degree of Exfoliation (%)
					*d*_003_	*d*_006_	Freeze Dried	Pre-Treated		
Cl^−^	0.20	0.18	2.55	3.082	8.182	4.074	24.495			
Cl^−^	0.25	0.25	3.16	3.066	8.026	3.999	24.036	24.304	810	15
Cl^−^	0.33	0.33	4.13	3.045	7.833	3.888	23.414			
I^−^	0.20	0.20	2.55	3.070	8.600	4.292	25.776			
I^−^	0.25	0.24	3.16	3.052	8.241	4.243	25.091		815	32
I^−^	0.33	0.32	4.13	3.045	------	------	-------			
CO_3_^2^^−^	0.20	0.20	2.55	3.077	8.021	3.987	23.993			
CO_3_^2^^−^	0.25	0.25	3.16	3.062	7.849	3.916	23.522	23.525	217	20
CO_3_^2^^−^	0.33	0.33	4.13	3.043	7.639	3.800	22.861			
SO_4_^2^^−^	0.20	0.21	2.55	3.068	8.589	4.292	25.760			
SO_4_^2^^−^	0.25	0.26	3.16	3.050	8.746	4.364	26.213	33.569	1066	15
SO_4_^2^^−^	0.33	0.34	4.13	3.029	8.843	4.414	26.510			

^1^*a* = 2*d*_110_; ^2^ *c* = (3*d*_003_ + 6*d*_006_)/2.

## Data Availability

Data sharing is not applicable to this article.

## Supplementary Materials

The following are available online at https://www.mdpi.com/1996-1944/14/2/346/s1, Figure S1: X-ray diffraction profiles of (003), (006) and (110) Bragg-reflections of as-synthesized, freeze dried Mg_1−*x*_Al*_x_*(OH)_2_(NO_3_)*x*·*m*H_2_O (*x* = 0.20, 0.25 and 0.33), Figure S2: Unit cell dimensions versus nominal composition *x* for Ni_1−*x*_Al*_x_*(OH)_2_(NO_3_)*_x_*·*m*H_2_O prepared at pH = 9.0 (■) and pH = 10.0 (■), Figure S3: Cuvettes with suspensions of exfoliated Mg_1−*x*_Al*_x_*(OH)_2_(NO_3_)*x*·*m*H_2_O after 4 h of sonication, Figure S4: Tapping mode AFM images of Mg_1−*x*_Al*_x_*(OH)_2_(NO_3_)*x*·*m*H_2_O, (a) *x* = 0.20, (b) *x* = 0.25, (c) *x* = 0.33 and its section profile analysis.
