# Supplementary material for: Efficient Exfoliation of Layered Double Hydroxides; Effect of Cationic Ratio, Hydration State, Anions and Their Orientations
Source: Materials (Basel). 2021 Jan 12;14(2):346. doi: 10.3390/ma14020346 (PMC7826757; doi:10.3390/ma14020346)
Supplement: Supplementary file 1 [file materials-14-00346-s001.pdf]

Supplementary information

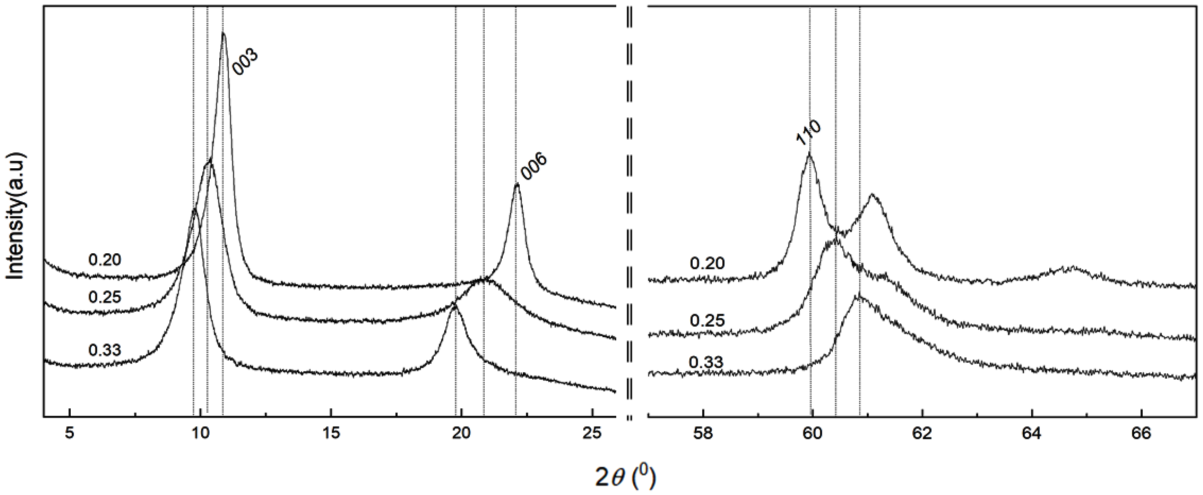

Figure S1. X-ray diffraction profiles of (003), (006) and (110) Bragg-reflections for as-synthesized, freeze dried $Mg_{1-x}Al_x(OH)_2(NO_3)_x{\cdot}mH_2O$ (x = 0.20, 0.25 and 0.33; λ = 1.540596Å).

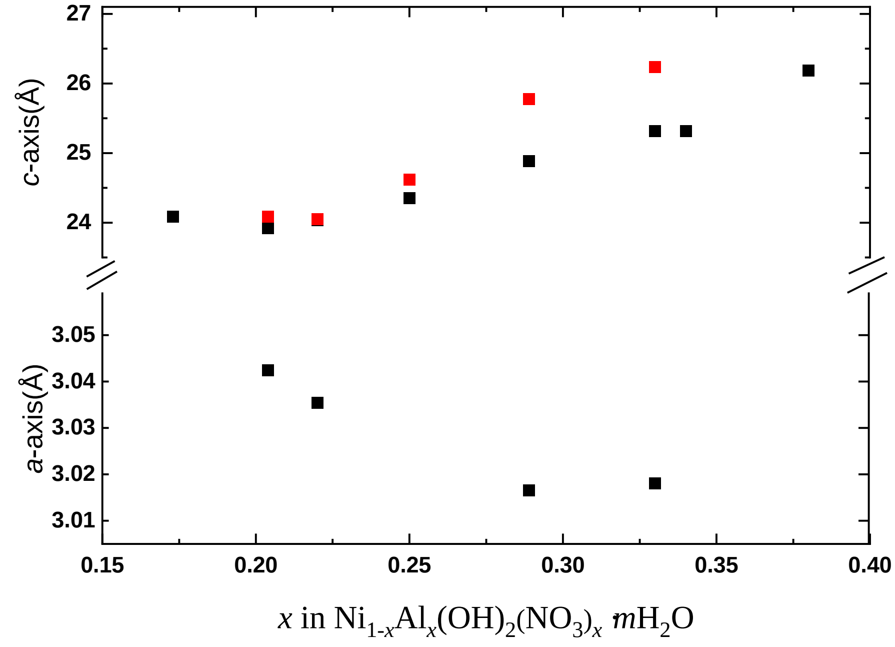

Figure S2. Unit cell dimensions versus nominal composition x for $Ni_{1-x}Al_x(OH)_2(NO_3)_x{\cdot}mH_2O$ prepared at pH = 9.0 (■) and pH = 10.0 (■).

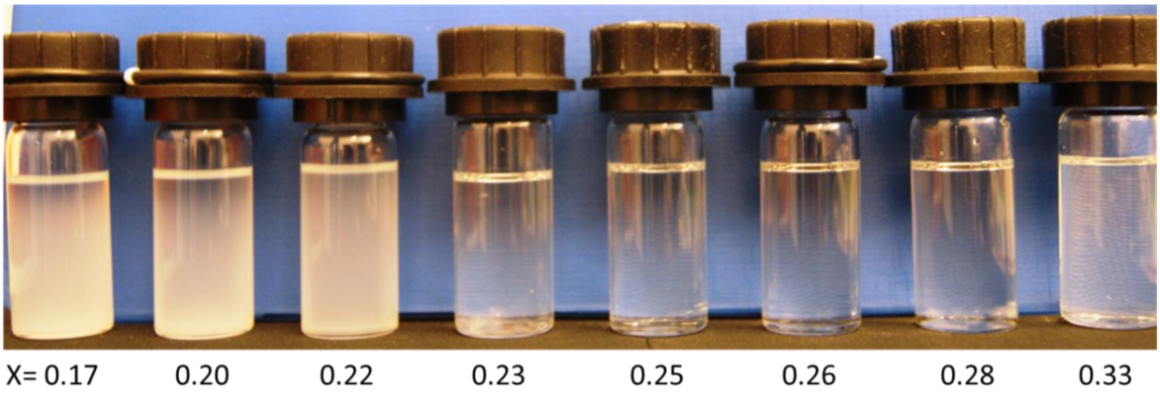

Figure S3. Cuvettes with suspensions of exfoliated $Mg_{1-x}Al_x(OH)_2(NO_3)_x \cdot mH_2O$ after 4 hours of sonication.

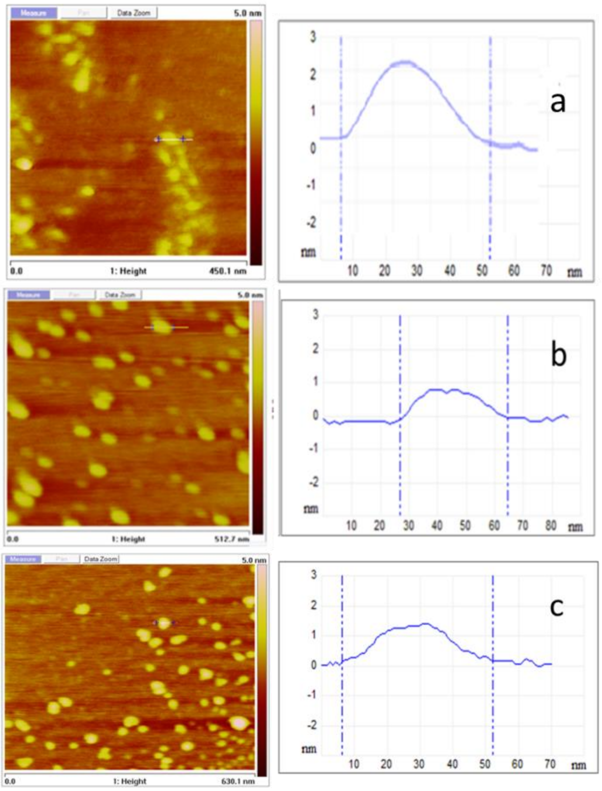

Figure S4. Tapping mode AFM images of $Mg_{1-x}Al_x(OH)_2(NO_3)_x \cdot mH_2O$, **a**) $x = 0.20$, **b**) $x = 0.25$, **c**) $x = 0.33$ and its section profile analysis.
